# Medicine

**Published:** 1892-06

**Authors:** J. Michell Clarke


### Progress of the Medical Sciences.

### MEDICINE.

The subject of the Cerebral Palsies of Early Life has been carefully considered by Drs. Sachs and F. Peterson, who base their observations upon an analysis of 140 cases,[1] of which 87 were males and 53 females. 105 were cases of hemiplegia, 24 of diplegia, and 11 of paraplegia. The paralysis was either congenital or developed in early life, arising (1) from disturbances during pregnancy or from a tedious or difficult labour; (2) after infectious diseases, during convulsions, or from causes not ascertained. Except in two cases, there was in all marked spasticity and extreme contractures; in all except one the reflexes were exaggerated, at any rate on the side of the paralysis: in all, growth was retarded; in some, athetosis and post-hemiplegic disorders of movement were present. All stages of mental impairment from weak-mindedness to complete idiocy were observed. A few patients showed good mental development. Sensibility and electrical reactions were unaffected. From an analysis of the cases, it appears that diplegia and paraplegia are more likely to be congenital, and hemiplegia to occur during the first four years of life; but 20 per cent. of cases of hemiplegia are congenital. In Osler's tables, 15 of 120 cases of hemiplegia were congenital. From the ages at which the patients came under observation, it is deduced that those affected with diplegia and paraplegia are short-lived, but this does not hold good for hemiplegia.

From a study of the circumstances attending the birth in 49 cases of congenital cerebral palsy, we learn that the forceps should be applied if necessary, or delivery hastened by other means, if protracted labour can be averted. As to the occurrence of monoplegia, the authors always found evidence, though it might be very slight, of affection of more than one limb. Circulation in the skin is often as sluggish, and the livid hue of the affected limbs as marked, as in infantile spinal paralysis. Epilepsy occurred in 44.3 per cent. of all cases. In 50 cases (nearly 50 per cent.) of the hemiplegics, it was

[1] *Trans. N. Y. Acad. Med.*, 1891, p. 183.

general in 41, of the Jacksonian type in 9, and consisted of attacks of *petit mal* in 1. In 80 cases there was mental impairment, in 52 per cent. of the hemiplegic, in 71 per cent. of the diplegic, and in 82 per cent. of the paraplegic patients. Idiocy was most marked in paraplegic cases (45 per cent.), and least in hemiplegic (6.75 per cent.). The authors think that though there are some considerations in favour of Strümpell's polio-encephalitis as the lesion in question in these palsies, its existence is not yet definitely proved, and should be diagnosed only in default of other causes. They conclude that the form of paralysis is not the most important factor in diagnosis; that acquired cases of hemiplegia and diplegia, especially the former, are apt to be due to the same causes that prevail in apoplexies in adults—namely, hemorrhage, thrombosis, and embolism—but that meningeal and cortical lesions are more frequent than in adults. Convulsions and loss of consciousness occur in all cases of cortical lesion, however slight, and in cases of sudden hemorrhage and embolism; the absence of convulsions at the onset probably indicates an intra-cerebral lesion; their occurrence points generally to a cortical lesion, or to embolism anywhere in the brain. In a few cases, affection of the lower centres of grey matter may also be attended with convulsions. Convulsions frequently repeated after the onset point with great certainty to a cortical lesion. Cases coming on after one of the acute infectious diseases have been proved to be due to vascular derangement, particularly embolism and hemorrhage, but some cases may arise from a polio-encephalitis corticalis. This is more probable if the onset is attended with high fever and convulsions. Traumatism is an important factor in the causation of meningeal hemorrhage during early life, and is particularly frequent during labour. Congenital cases of hemiplegia and diplegia may be due to an early meningeal hemorrhage, or possibly to an early encephalitis. In acquired and congenital cases of hemiplegia and diplegia there may be either porencephalus or a simple arrest of development. If acquired, there would seem to be a history of slow development of symptoms, without predisposing cause. If congenital, there may be a history of traumatism to the mother. In later life, hemiplegia or diplegia associated with a large degree of mental impairment and with contractures points to a general cortical atrophy, sclerosis with secondary degenerations, or to a condition of porencephalus the origin of which it is often difficult to determine.

Concerning the cerebral atrophies of childhood, with especial reference to the operation of craniotomy for imbecility, epilepsy, and paralysis, Dr. Allen Starr[1] says that craniotomy, or the operation of producing an opening of some extent in the skull, with the object of relieving pressure on the brain or in some unknown manner of stimulating its development, has been proposed and performed in certain cases, which fall into three groups: (1) those of hemiplegia with or without athetosis; (2) cases of mental defects of various grades; (3) cases of sensory defects of different types. Epileptiform seizures of *petit* or *grand mal* type occur frequently in patients who may be assigned to any one of these groups, and may be the particular symptom with regard to which advice is sought. The chief clinical facts of importance in the first group are the time and character of the onset, the degree of spontaneous recovery, and the question whether the epilepsy is of so severe a type as to be dangerous. (See above paper for nature of lesions in these cases.) In the second group, the symptoms are mental rather than somatic:—slowness in learning to talk, want of reasoning power, thought, judgment, or self-control; constant and generally mischievous restlessness. Some children may be bright and precocious, but there is an absence of moral ideas; some are dirty and imbecile. These cases remain incapable of supporting themselves or directing their conduct throughout life, and have to be cared for. Cases of the third group are more rare; they have no motor or mental defects, though they may be the subjects of epilepsy. Many cases of deaf-mutism probably belong to this class.

With regard to the question of operation—the disease is at a standstill, is not dangerous to life, and an operation is not free from danger. It is to be remembered that the brain is capable of growth and development up to the age of 14. Can such development be aided by an operation? Many of the pathological conditions present are such as to admit of operative relief. Study of the pathology shows that the varying symptoms are due to the varying situation rather than to the varying nature of the lesion; the various pathological processes present lead to sclerosis with atrophy of the brain. In clinical cases of the first group, the sclerotic atrophy involves the Rolandic area and motor tracts, and generally the basal ganglia as well. In the second group, the anterior convolutions, or sometimes the entire hemisphere to a greater or less extent, and in the third group the posterior and lateral parts of the hemispheres are involved. In many cases the epileptic attacks are so frequent that any risk might be taken, provided that they could be stopped.

[1] *Med. Rec.*, Jan. 23, 1892.

Dr. Starr concludes that (1) hemiplegia, sensory defects, and imbecility, with or without epilepsy, in children are chronic diseases, incurable by medical treatment. Any means which may be legitimately used to save the patient from a life of invalidism and take the burden of his case from his family are to be employed. (2) The pathological conditions may be either gross defects and atrophies of the brain, or an arrest of development in the cerebral cells, without any naked-eye changes. (3) The pathological condition existing cannot at present be determined without an exploratory operation. (4) Such operations are not without danger, but if made as short as possible, and caution used in opening the dura mater, the dangers may be avoided. (5) When manifest atrophy is present, the result of operation is *nil*. When there is arrested development of the cerebral tissue, it may be useful. When clots, cysts, or tumours are found and removed, the chance of recovery is increased. When the skull is markedly microcephalic from early union of the sutures, the increased space given to the brain by the operation appears to stimulate its growth and development. (6) Epileptic attacks are often reduced in frequency and modified in character by craniotomy. When the opening in the skull remains covered over only by the soft tissues, it appears to act as a safety-valve, allowing changes in the intra-cranial contents to occur without producing pressure upon the brain. (7) While hemiplegia, athetosis, aphasia, and sensory defects have been relieved by operation, it is as yet impossible to predict to what extent imbecility may be thus relieved. (8) Reports of cases should be made in full, and not within six months of the time of operation, as conclusions cannot be trustworthy unless arrived at by long observation.

Dr. B. Sachs [1] thinks there is reason to doubt whether the unsuccessful cases of treating epilepsy surgically have been reported with sufficient candour and promptness, and asks the question whether, if the attacks are inhibited for weeks or months, this temporary success is to be put to the credit of the operation, seeing that in 1875 Maclaren pointed out that there was apt to be temporary improvement after any operation of whatever sort. Probably some of the supposed cures after ovariotomy may occur in this way. Epilepsy being a symptom only, great importance is to be attached to the discovery of the cause which exists in many cases, *e.g.* a

[1] *New York Med. Journ.*, Feb. 20, 1892.

long-forgotten accident or injury. Féré and Chaslin, in five cases of epilepsy, found in all increase of neuroglia tissue, a secondary sclerosis of the cortex associated with epilepsy.[1] In idiopathic epilepsy the initial causal lesion is not determined, but in cases of Jacksonian epilepsy—in which we operate—due to focal disease, of traumatic or other origin, the focus of disease is present, together with a secondary sclerosis. In time the focal lesion may disappear, but the secondary sclerosis can be recognised. In early childhood meningeal hemorrhage may give rise to secondary changes (lobar sclerosis) in the cortex; the same process occurs in traumatic meningeal hemorrhage. The chief symptoms are paralysis and epilepsy. This secondary sclerosis may be the source of irritation, and the diseased area constitute a discharging centre; the neglect of careful microscopical investigation of the cortex accounts for the absence of the demonstration of sclerosis in cases of traumatism. There is thus the initial focal lesion and the secondary sclerosis to be dealt with: we have to prevent the development of secondary sclerosis or to neutralise its effects. For the first indication, we can only, in our ignorance of the exact conditions of the development of sclerosis, act by removing or diminishing the initial lesion, which means an early operation in cases of traumatic or organic disease. The only known method of fulfilling the second indication is by excision to rid the brain of the sclerosed areas. Excision means loss of the functions of the excised centre; but this is often recovered from in young subjects, and most patients would prefer paralysis to epilepsy.

*Practical Conclusions.*—(1) In a given case of traumatic or organic lesion, operate as early as possible, to prevent the development of secondary sclerosis. (2) Onset of epilepsy is a warning that sclerosis has been established; if not before, operate now, to avoid an increase of this trouble. (3) Excision is the only rational operation, and if other centres are not in an irritable condition it may be completely successful. Traumatic cases are of immediate interest; if there is any doubt as to depression of bone, it is best to trephine, to make sure. In cases of traumatic epilepsy, in which there is no old scar and no depressed bone, and yet improvement follows operation, the improvement is probably to be attributed to the relief of increased pressure due to adhesions between the

[1] The reader may be referred to Bevan Lewis's researches on the morbid changes in the cortex in epilepsy. *A Text Book of Mental Diseases*, pp. 522–528.

brain and its coverings, or to traumatic cysts. The trephine opening should be made as large as possible. The drawbacks to operation lie in the paralysis which results after the excision of cortical centres, and to failure of the operation from the fact that secondary sclerosis has spread to neighbouring cortical areas, which have thus become discharging centres. The cicatrix of the operation may also have an injurious effect on the cortex. The author considers that the cerebral palsies of children, complicated with epilepsy, are the cases of non-traumatic epilepsy, which demand surgical interference. Simple trephining is more often successful in these than in traumatic cases, on account of the frequency with which cysts occur. Excision of cortical centres is also less serious in children, because other portions of the cortex may take on the functions of the part removed. He thinks that if these cases of infantile cerebral palsy were more generally recognised and the tendency to epilepsy checked, the total number of epileptics would be notably diminished.

Dr. Hayes Agnew, whose death we have now unfortunately to lament, had, in the *University Medical Magazine* for October, 1891, a paper on "The Present Status of Brain Surgery," based on the experience of Philadelphia Practice. In 57 cases trephining was done for traumatic epilepsy. Of these 57 cases, 41 recovered from the operation, 4 died, while in the remaining 12 the result is unknown. The ages varied from 7 to 49 years. The mortality did not exceed 7 per cent. 32 cases experienced temporary benefit; 9 obtained no relief; 4 passed out of observation; 4 were operated on too recently to express any opinion; 4 were cured, and 4 died. The author concludes that traumatic epilepsy is practically incurable by surgical operations, and that a considerable number of such cases had better be relegated to the domain of pure medicine. But he believes that a certain number of patients in this class, with whom internal remedies have no controlling influence on the fits, may with propriety be operated on as a palliative measure. He assumes that surgery is responsible for the great majority of traumatic epileptics, and the old doctrine that depressed fractures of the skull without symptoms require no operative interference has, in his opinion, been the cause of very many of the unfortunate sequelæ of head injuries.

M. Chas. Audry reports the interesting case[1] of a little boy of seven months, who had a spina bifida, in the sac of which an ulcerated opening formed, which was followed by

[1] *Prog. Méd.*, Feb. 27, 1892.

the discharge of clear fluid and subsequently of pus, after which the opening closed. Soon afterwards the head began to enlarge, and an enormous chronic hydrocephalus developed. An attempt to reopen the sac of the spina bifida failed, and the skull was then trephined in the right parietal region and a horsehair drain passed into the ventricle. Death took place some hours later, the child's temperature being 105.8° F. In his remarks on the case the author quotes two cases of hydrocephalus by A. Broca, [1] in which trephining and draining of the ventricles was practised. Other cases alluded to are three by Keen: in one the operation was performed for dropsy of the ventricle, due to a cerebellar tumour, and death occurred after 45 days; in the second, of chronic hydrocephalus, death took place on the fifth day; the third patient, who was suffering from tubercular meningitis and was operated on *in extremis*, lived four hours. Mayo Robson, in the case of a child with suppurating otitis and paresis of the arm, in addition to other cerebral symptoms, trephined over the arm centre, and not finding pus, punctured the lateral ventricle; recovery took place. In another case of hydrocephalus, following the cure of a spina bifida by injection of Morton's fluid, he drained the ventricles, and death occurred on the seventh day. M. Phocas, who has also reported one case in which he performed this operation, lost his patient at the end of eight days. Out of six cases in which trephining with drainage of the ventricles has been performed for chronic hydrocephalus, five have therefore died.

J. MICHELL CLARKE.

